# Evaluation of radiological and anatomical features of skull bones in adult Saanen goat

**DOI:** 10.1002/vms3.1435

**Published:** 2024-03-29

**Authors:** Siamak Alizadeh, Pourya Kamfar, Mohammadreza Hosseinchi

**Affiliations:** ^1^ Department of Clinical Sciences Faculty of Veterinary Medicine Naghadeh Branch Islamic Azad University Naghadeh Iran; ^2^ Faculty of Veterinary Medicine Urmia Branch Islamic Azad University Urmia Iran; ^3^ Department of Basic Sciences Faculty of veterinary medicine Urmia Branch Islamic Azad University Urmia Iran

**Keywords:** anatomy, radiology, Saanen goat, skull

## Abstract

**Background:**

Obtaining accurate knowledge of the anatomical structure of the skull helps in ontogenetic studies and determining sexual polymorphisms.

**Objectives:**

This study uses anatomical dissection and radiography to develop a size standard for the skull of the Saanen goat; information that will be applied to clinical evaluation and decision‐making.

**Methods:**

A total of 14 adult Saanen goat skulls (7 male and 7 female goats) were taken from the slaughterhouse and transferred to the clinic of the Faculty of Veterinary Medical Teaching Hospital, Urmia University, Urmia, Iran. Radiographs of each skull were prepared in the dorsal–ventral, left and right lateral recumbency. These heads were then brought over to the anatomy department, where the chosen morphometric traits were assessed and their means recorded.

**Results:**

According to the findings of this study, the dorsal view of the skull revealed an incisive raphe and a widespread foramen of the nose. In the ventral view, the incisive bones were observed in the most cranial region of the skull. The base of the occipital bone was relatively large, and the jugular process was well‐developed. In the lateral view, the incisive bone was extended to the cranial and had a narrow process on the dorsal surface. Regarding infra‐orbital foramen, it was visible in the maxillary bone. Moreover, the lacrimal bone was perceived as a distinct bone.

**Conclusion:**

The precise standards acquired in this study can be utilized to interpret the findings and make clinical decisions about the normal and abnormal size of the bones that make up the skulls of the Saanen goats.

## Graphical abstract

The current study aimed to evaluate the anatomical and radiological structure of the Saanen goat skull. The precise standards acquired in this study can be utilized to interpret the findings and make clinical decisions about the normal and abnormal size of the bones that make up the skulls of the Saanen goats.

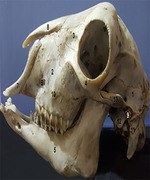

## 1 INTRODUCTION

The Saanen goat is an important milk‐producing goat breed with a high reproductive yield (Sadjadian et al., 2013). Saanen goats reared under good care and feeding conditions can provide a total of 700–900 L milk during 2.5‐year period (Gökdai et al., 2020). However, the goats may be exposed to injury in the head and skull regions. Identifying how different parts of the complex structure of the head are interconnected may help in the treatment of fractures (Tohidifar & Masoudifard, 2020). In addition, anatomical features of the skull might be influenced by genetic and environmental factors that help in interpreting differences between and within breeds or species (Masoudifard et al., 2022). Obtaining accurate knowledge of the anatomical structure of the skull helps in ontogenetic studies and the determination of sexual polymorphisms (Olopade, 2018). Imaging systems are valuable to identify different parts and increase and update the knowledge of head anatomy.

Skull morphometry has been evaluated in several species, including wild species (Erdam, 2016), domestic animals (Hykaj et al., 2012; Poul, 2010), farm animals (Parés‐Casanova et al., 2020), Iranian native goats (Monfared & Sheibani, 2013), Black Bengal goat (Uddin et al., 2019) and Markhoz goat (Goodarzi, 2014). Frake (2008) evaluated frontal sinuses and head‐butting in goats and showed differences in various species. Choudhary and Singh (2016) evaluated morphological studies of the cranial cavities in Indian goats and showed significant differences between species. Tohidifar and Masoudifard (2020) evaluated the anatomy of the head in the Saanen goat using computed tomography and reported a lack of palatine and sphenoidal sinuses in the Saanen goat.

Whether radiographic images of the Saanen goat skull can be effective in identifying its anatomical features and whether this diagnostic imaging method can play an important role in diagnosing bone diseases in this area form the core of this research. Thus, this study aims to evaluate the anatomical and radiological structure of the Saanen goat skull and also to determine a precise standard for the size of the bones that make up this area, which may thus be utilized to interpret clinical findings and inform clinical decisions.

## 2 MATERIALS AND METHODS

### 2.1 Study design and animals

A total of 14 adult Saanen goat skulls (7 male and 7 female goats) were obtained from the slaughterhouse and transferred to the clinic of the Faculty of Veterinary Medical Teaching Hospital, Urmia University, Urmia, Iran. The mean age of these slaughtered goats was 2.7 years and their average weight of 31.3 kg. The type of X‐ray device used to prepare radiographs was a Dean 44 X‐ray machine made in South Korea, and the applied kVp and mAs were 45 and 3.6, respectively.

### 2.2 Description of the method

Radiographs of each skull were performed in dorsal–ventral, ventral–dorsal and left and right lateral recumbency. Following radiography, the skin and muscles of the head area (to the bones) were removed using a scalpel blade. Then the skull bones were washed with water, placed in potassium hydroxide 10% for 5 days and finally bleached with H_2_O_2_. After that, each skull was exposed to sunlight for a week to dry out (Vistro et al., 2015). We examine each bone of the skull separately in terms of morphological characteristics. The morphometric indices were measured, and their means were recorded as follows:

Condylobasal length (Cb.L), braincase width (Bc.W), muzzle width (Mu.W), zygomatic width (Zg.W), orbital diameter (Or.D), maxillary tooth‐row length (Mt.L), ramus mandibular height (Rm.H), mandible length (Md.L), mandibular tooth‐row length (Mdt.L) and coronoid height (Co.H) (Table 2).

### 2.3 Statistical analyses

Descriptive statistics analyses were done using the SPSS package. The data were described using the mean, std. deviation and std. error mean.

## 3 RESULTS

### 3.1 Morphologic results: dorsal view of the skull

The skull in the dorsal view: From cranial to caudal, nasal bones, premaxilla, maxilla, lacrimal bone, frontal bone, zygomatic bone, parietal bone, squamous part of the temporal bone, interparietal bone and upper region of occipital bone are observable. We identified an incisive raphe in the cranial position. The nasal external foreman is wide and is seen in the cranial section. The nasal conchae were spread towards the cranial region of the external foramen and are observable in the dorsal region. The nasal bones stretch in the form of two strips between the two sections of the frontal bone.

**FIGURE 1:**
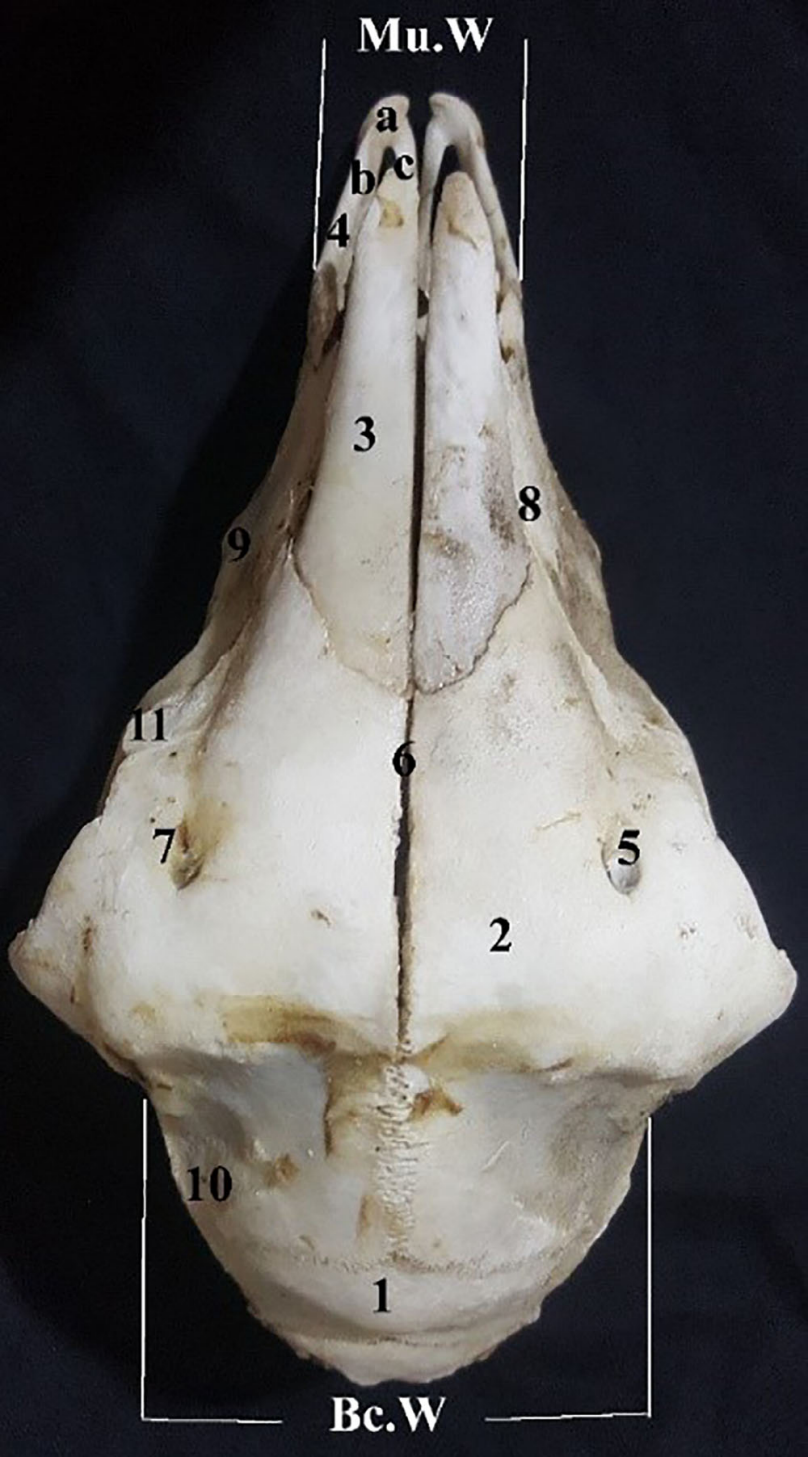
Dorsal view of the skull: (1) parietal, (2) frontal, (3) nasal, (4) incisive bone (a: alveolar process, b: nasal process, c: hard palate process), (5) supraorbital foramen, (6) interfrontal suture, (7) supraorbital fissure, (8) maxilla, (9) facial tuberosity, (10) squamous part of temporal bone, (11) lacrimal bone. Bc.W, braincase width; Mu.W, muzzle width.

The cranial region of the nasal bone joins with the premaxilla and interparietal bones. The nasal process of the interparietal bone extends towards the frontal bone. The prelacrimal crest is seen in the cranial part of the lacrimal foramen. The maxilla is a big bone whose dorsal‐caudal part is more developed. The infra‐orbital foramen seen under the orbit was opened in the space between the third premolar teeth and the first molar teeth. The cranial part of the parietal bone is concave. The zygomatic arch is horizontal and formed by the zygomatic bone and the zygomatic process of the maxilla and a part of the temporal bone. The zygomatic process of the maxilla is wide and rises from above the first molar teeth and joints to the zygomatic bone. The zygomatic bone is present in bar form in the median region of the zygomatic arch. The interparietal bone is positioned between the cranial portion of the parietal bone and the occipital bone (Figure 1).

### 3.2 Ventral view of the skull

**FIGURE 2:**
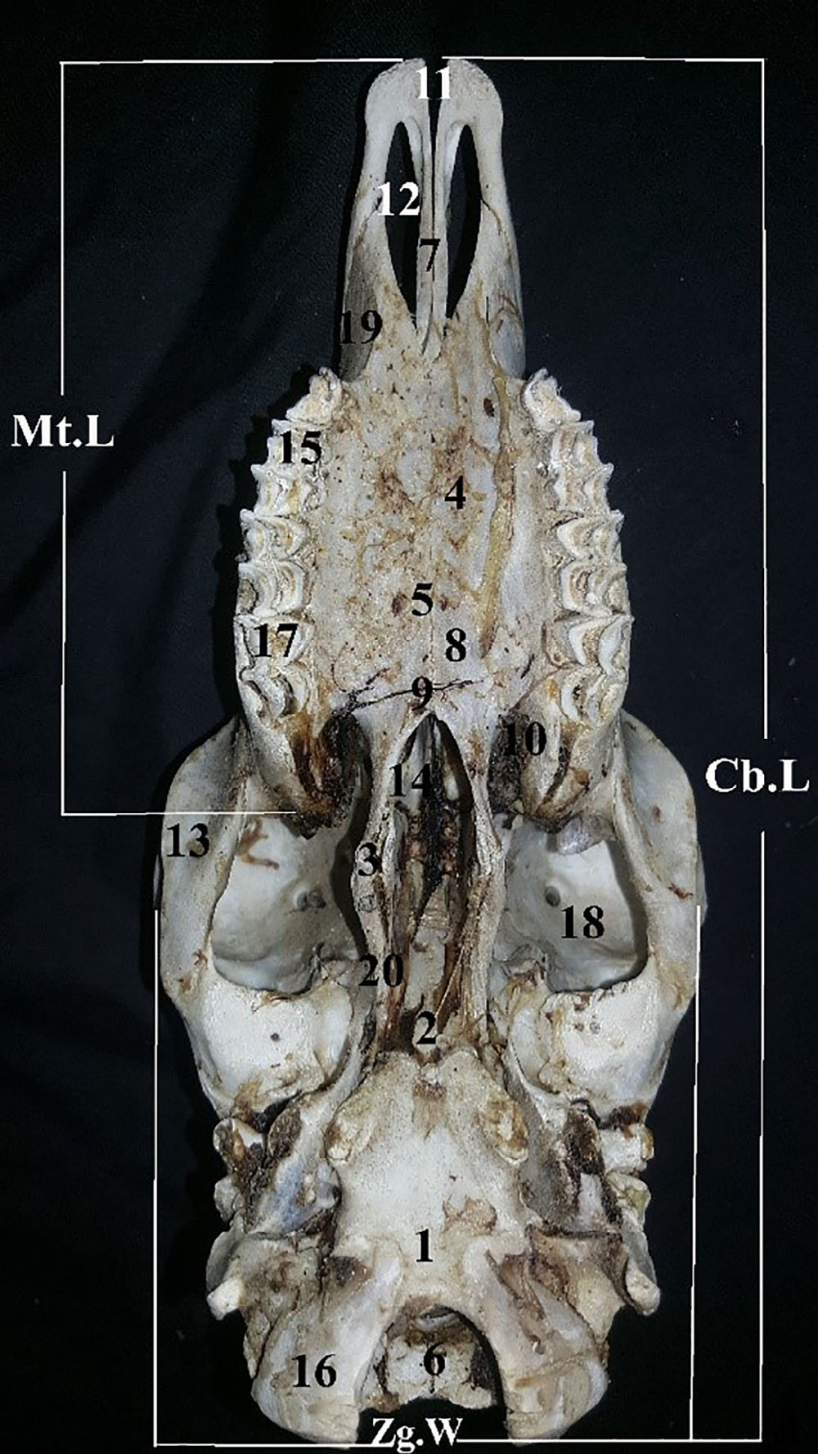
Ventral view of the skull: (1) occipital bone, (2) basisphenoid bone, (3) pterygoid hamulus, (4) palatine process of maxilla, (5) minor palatine foramen, (6) foramen magnum, (7) nasal process of maxilla, (8) palatine, (9) wing of vomer, (10) pterygoid fossa, (11) interincisive notch, (12) palatine fissure, (13) maxilla bone, (14) presphenoid bone, (15) premolar teeth, (16) occipital condyle, (17) molar teeth, (18) ethmoidal foramen, (19) interalveolar space, (20) wing of basisphenoid. Cb.L, condylobasal length; Mt.L, maxillary tooth‐row length; Zg.W, zygomatic width.

In the ventral view of the skull from cranial to caudal, the premaxilla, maxilla, palatine and presphenoid bones are observable. The premaxilla (incisive bone) is seen in the closet the region of the skull. An interincisive notch is positioned between the incisive bone and the maxilla. The hard palate is formed by the incisive bone, maxilla and palatine bones. The palatine bone has a large greater palatine foramen. The transverse crest is found in the caudal region of the palatine. The bone is extended and forms an intrapterygoid fossa. The pterygoid external and internal processes are positioned on the sides. There are small processes on the sphenoid bone. The oval fossa is positioned cranial to the tympanic bulla. The occipital bone is big, and the jugular process is well‐grown and has caudal‐laterally oriented (Figure 2).

### 3.3 Lateral view of the skull

In this view, the teeth, premaxilla, maxilla, lacrimal, frontal, zygomatic arch, parietal and occipital bones are visible. The premaxilla bone is cranial direction and has a narrow process in the dorsal view. The infra‐orbital foramen is observable in the maxilla. The zygomatic arch is observed as well.

The tympanic part of the temporal bone articulates caudally with the mastoid process, and its cranial edge is prominent and plays a role in the creation of the occipital crest.

**FIGURE 3:**
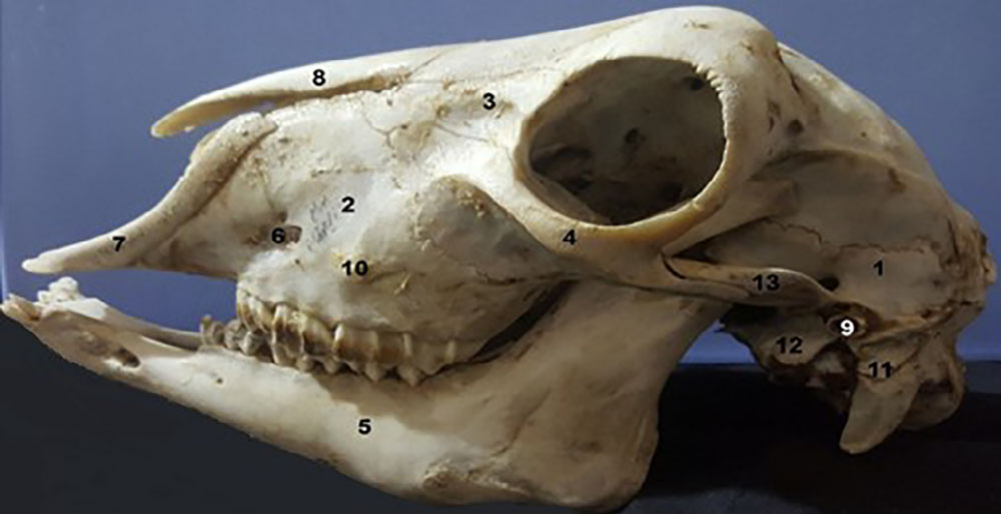
Bones in lateral position: (1) temporal, (2) maxilla, (3) lacrimal, (4) zygomatic, (5) mandible, (6) infra‐orbital foramen, (7) incisive, (8) nasal, (9) external acoustic meatus, (10) facial tuberosity, (11) mastoid process, (12) tympanic bulla, (13) zygomatic process.

**FIGURE 4:**
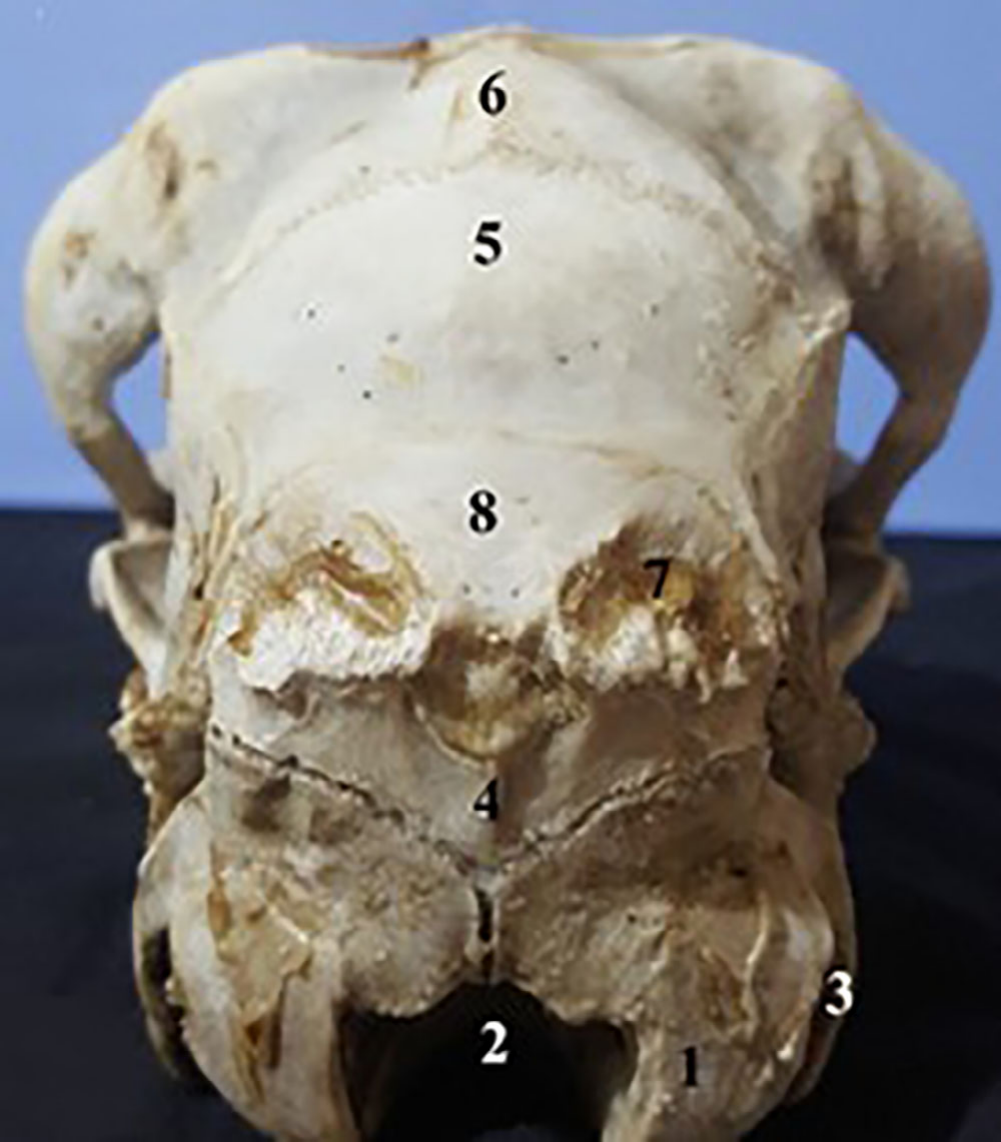
Bones in caudal position: (1) occipital condyle, (2) foramen magnum, (3) jugular process, (4) external occipital crest, (5) parietal bone, (6) frontal bone, (7) squamous part of occipital, (8) occipital bone.

The lacrimal bone, part of the maxilla and frontal, forms a lateral section of the orbit. The lacrimal bone is a distinct bone in this view. In this view, the lacrimal foramen, lacrimal crest and infra‐orbital foramen are observable. The external acoustic meatus is observable in the tympanic part of the temporal bone (Figure 3). In caudal view of the skull, the jugular processes of the occipital bone are visible. The occipital condyle and jugular process are observable on the sides of the foramen magnum. The zygomatic arch and tympanic bulla are observable in the lateral position. The mandible and all the bones are seen in the lateral view of the skull (Figure 4; Table 1).

**TABLE 1:** Saanen goat skull bones in anatomical and radiological evaluation in ventrodorsal, dorsoventral and lateral views.

Bone	View		
	Dorsoventral	Ventrodorsal	Lateral
**Nasal**	‐ The nasal external foreman is wide ‐ Jointed with the premaxilla and interparietal bones ‐ Nasal process is extends toward the frontal bone	−	−
**Maxilla**	‐ The maxilla is a big bone whose dorsal‐caudal part is more developed	−	‐ The infra‐orbital foramen is observable in the maxilla
**Lacrimal**	‐ Make the orbital bone ‐ The prelacrimal crest is seen in the cranial part of the lacrimal foramen	−	‐ The lacrimal bone, part of the maxilla and frontal form a lateral section of the orbit ‐ Is a distinct bone in this view
**Zygomatic**	‐ The zygomatic arch is horizontal ‐ The zygomatic bone is present in bar form in the median region of the zygomatic arch	−	‐ The zygomatic arch is observable with its forming bones ‐ The zygomatic arch and tympanic bulla are observable in this view
**Parietal**	‐ The cranial part of the parietal bone is concave	−	−
**Interparietal**	‐ The interparietal bone is positioned between the cranial portion of the parietal bone and the occipital bone	−	−
**Incisive**	‐ An incisive raphe is seen in the cranial position	‐ Is seen in the closet the region of the skull ‐ An interincisive notch is positioned between the incisive bone and the maxilla ‐ The hard palate is formed by the incisive bone, maxilla and palatine bones	‐ The premaxilla bone is cranial direction and has a narrow process in the dorsal view
**Palatine**	−	‐ The palatine bone had a large major‐palatal foramen	−
**Pterygoid**	−	‐ The pterygoid external and internal processes are positioned on the sides	−
**Occipital**	−	‐ The occipital bone is big and jugular process is well‐grown and has a caudal‐laterally oriented	−

**TABLE 2:** The results of morphometric measurement of Saanen goat skull (mm).

No.	Rm.H	Co.H	Mdt.L	Md.L	Mt.L	Or.D	Zg.W	Mu.W	Bc.W	Cb.L
**1**	21.6	27.8	31.7	61.4	42.4	21.2	47.8	85.1	40.8	85.2
**2**	21.6	27.3	31.5	61.1	42.3	20.6	48.1	84.7	41.2	85.6
**3**	21.8	28.1	32.2	60.6	41.9	20.8	48.6	85.6	40.0	85.4
**4**	22.0	27.8	31.4	61.8	42.5	21.4	46.9	85.4	41.4	84.9
**5**	21.5	27.0	31.6	61.6	42.4	21.6	46.5	85.8	41.5	86.1
**6**	21.5	28.1	31.3	62.1	42.2	21.0	47.1	85.1	41.9	85.5
**7**	21.2	28.2	31.8	61.8	42.4	22.8	47.1	84.8	42.2	86.6
**8**	21.5	27.6	30.9	61.3	42.6	21.3	48.2	85.1	42.3	84.8
**9**	22.2	28.8	32.1	62.1	42.6	22.1	48.9	85.4	41.8	86.6
**10**	21.5	27.9	30.5	61.7	41.9	21.9	47.8	85.7	42.7	85.7
**11**	21.4	27.7	31.2	60.2	41.7	20.1	48.7	85.4	40.0	85.5
**12**	21.7	27.5	31.6	61.3	42.5	20.7	48.1	85.5	41.1	85.3
**13**	21.4	28.0	32.2	62.0	42.2	21.9	48.3	84.2	40.9	86.8
**14**	21.3	27.9	31.2	61.8	42.8	21.3	47.8	85.3	41.7	85.4
**Mean**	21.5	27.8	31.5	61.4	42.3	21.3	47.8	85.2	41.3	86.6
**SD**	±2.04	±4.2	±4.47	±3.3	±4.12	±1.66	±2.31	±2.19	±2.34	±6.54

Abbreviations: Bc.W, braincase width; Cb.L, condylobasal length; Co.H, coronoid height; Md.L, mandible length; Mdt.L, mandibular tooth‐row length; Mt.L, maxillary tooth‐row length; Mu.W, muzzle width; Or.D, orbital diameter; Rm.H, ramus mandibular height; Zg.W, zygomatic width.

### 3.4 Lateral view of the mandible

**FIGURE 5:**
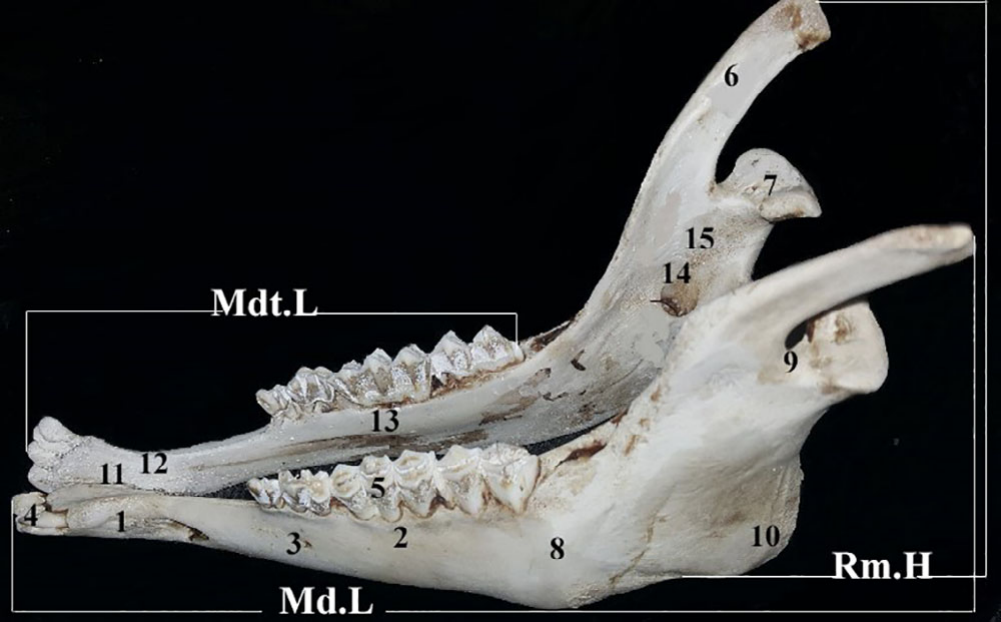
Lateral view of mandible: (1) incisive part, (2) molar part, (3) mental foramen, (4) incisor teeth, (5) molar teeth, (6) coronoid process, (7) condyloid process, (8) body of mandible, (9) mandibular notch, (10) angle of mandible, (11) lingual surface, (12) incisive part, (13) molar part, (14) mandibular foramen, (15) ramus. Rm.H, maximum mandibular height; Md.L, mandible length; Mdt.L, mandibular tooth‐row length.

The cranial section of the coronoid process forms an angle of 90° with the body and is curved at the end. The coronoid process has distinct crests in the lateral section. The caudal section of the crest fossa was extended. The condylobasal region extends into the dorsal–ventral region. The mental foramen is observable in the ventral region cranial to the second pre‐molar (Figure 5).

### 3.5 Medial view of the mandible

The symphysis joins the left and right mandibles and is in the cranial‐ventral section of the mandibular body.

The obtained dental formula was as follows:

$$2\times \left(I0/4\;C0/0\;\mathrm{Pm}3/3\;M3/3\right)=32$$

### 3.6 Radiology results

**FIGURE 6:**
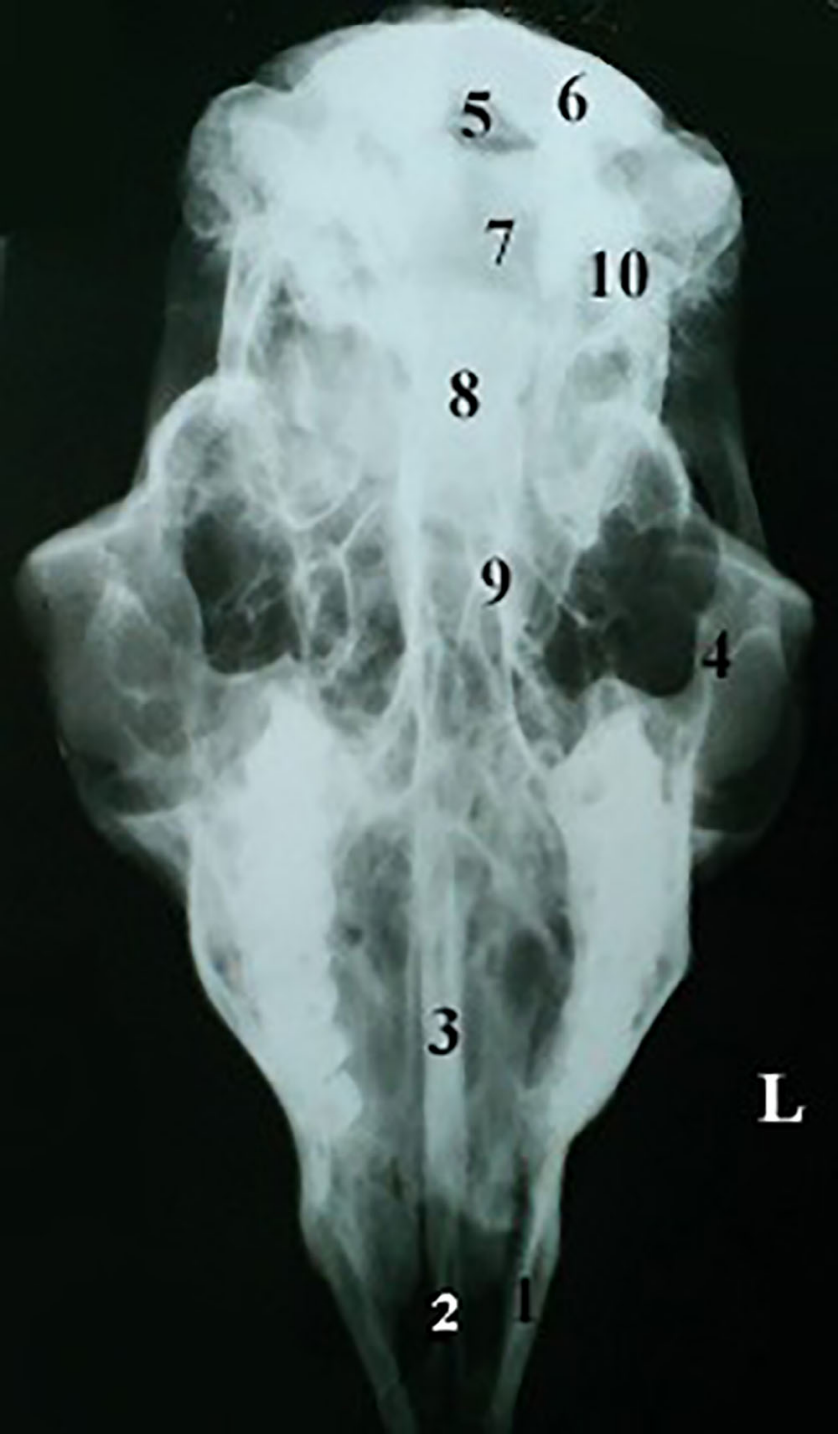
Dorso‐ventral view of the skull and mandible. Skull: (1) incisive bone, (2) palatine fissure, (3) vomer, (4) maxilla bone, (5) foramen magnum, (6) occipital condyle, (7) occipital bone, (8) basisphenoid bone, (9) pterygoid hamulus, (10) tympanic bulla. L, left side.

In dorsal–ventral view, in addition to the structures seen examination by the naked eye in the same view, the tympanic part of the temporal bone, occipital bone and the mandibular bone parts, including the body and condylar and rostral processes, are also visible (Figure 6).

**FIGURE 7:**
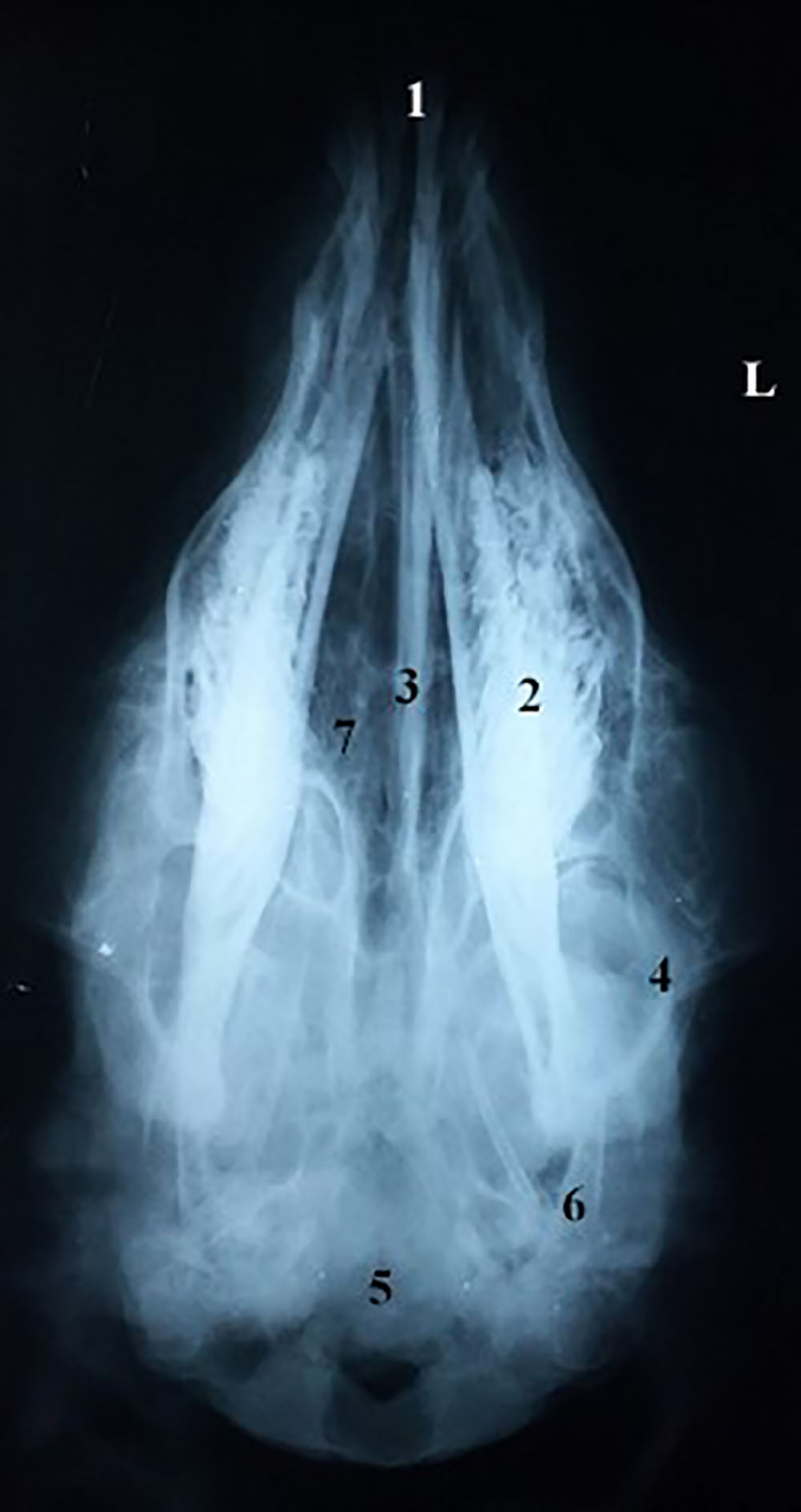
Ventral–dorsal view of the skull: (1) incisive teeth, (2) molar teeth, (3) nasal membrane, (4) zygomatic arch, (5) occipital bone, (6) coronoid bone, (7) hard palate. L, left side.

**FIGURE 8:**
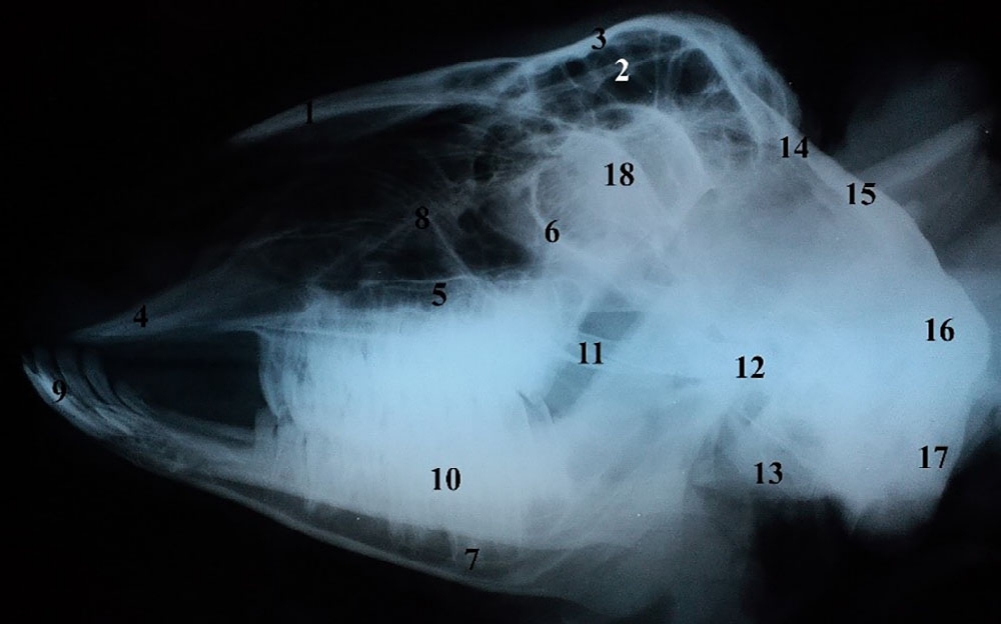
Right lateral view of the skull radiograph: (1) nasal bone, (2) frontal sinus, (3) frontal bone, (4) incisive bone, (5) maxilla bone, (6) ventral border of orbit, (7) mandible, (8) nasal concha, (9) incisor teeth, (10) molar teeth, (11) zygomatic arch, (12) temporal joint of mandible, (13) external acoustic meatus, (14) frontal, (15) parietal, (16) occipital bone, (17) occipital condyle, (18) optic canal.

In the ventral–dorsal view, the teeth, nasal bone, premaxilla or incisor bone, maxilla, body and processes of the mandible, that is condylar and coronoid processes, zygomatic arch, frontal, tympanic bulla and occipital bone, were observed (Figure 7). In the lateral view, structures such as the nasal bone, frontal sinus, frontal bone, incisive bone, maxilla bone, the ventral border of orbit, the body of mandible, nasal concha, incisor teeth, molar teeth, zygomatic arch, the temporal joint of the mandible, external acoustic meatus, frontal bone, parietal bone, occipital bone, occipital condyle and optic canal were seen (Figure 8).

**FIGURE 9:**
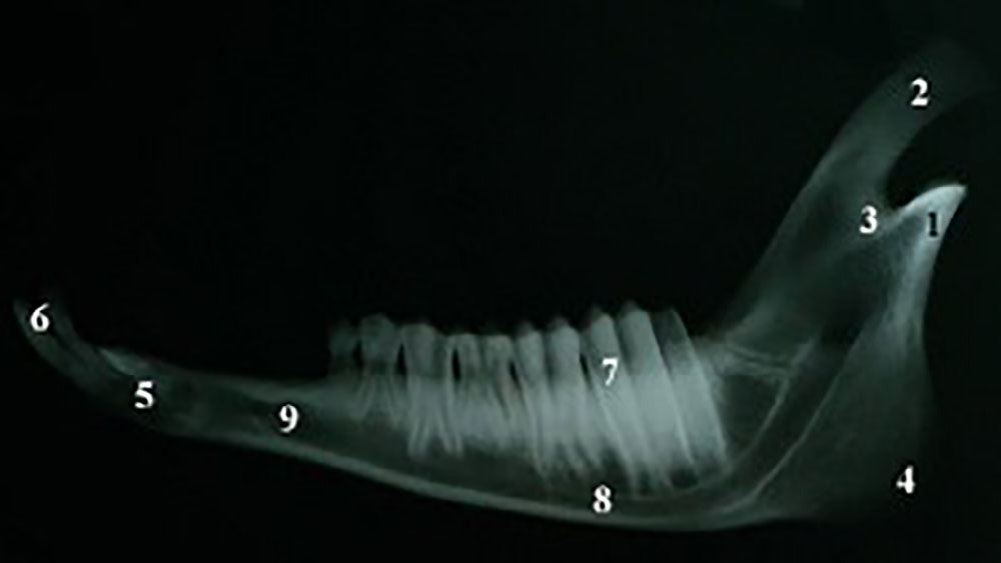
Lateral view of the mandible: (1) condylar process, (2) coronoid process, (3) mandibular notch, (4) angle of mandible, (5) incisive part, (6) incisor teeth, (7) molar teeth, (8) body of mandible, (9) mental foramen.

In the lateral view of the mandible, the coronoid and condyloid processes, the mandibular notch, angle of mandible, the premaxilla, incisor teeth, molar teeth, the body of the mandible and mental foramen were observed (Figure 9). In Box plot 1, some different traits (indices) of Saanen goats are compared with other goat breeds.

**Box plot 1:**
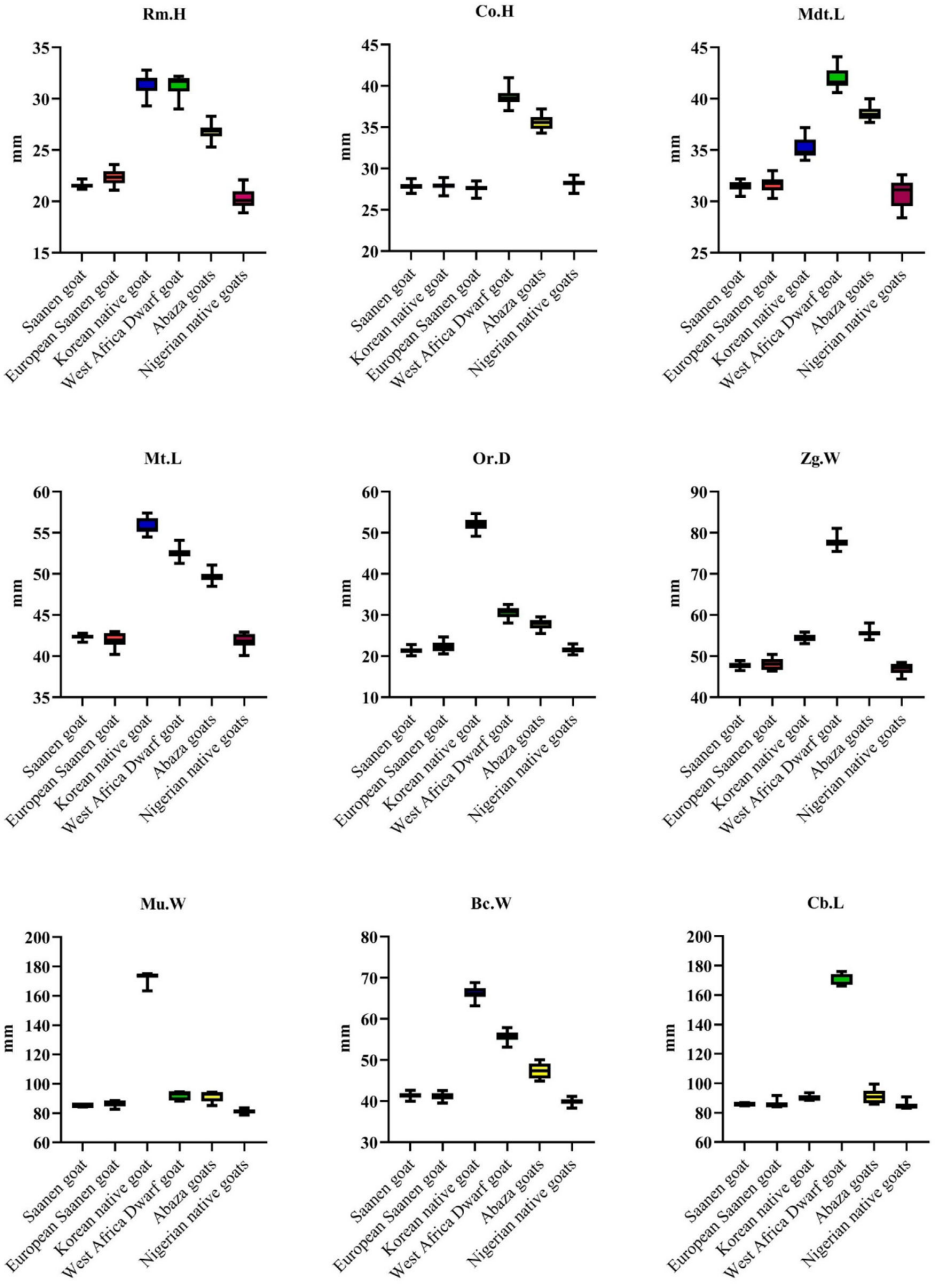
Comparison of condylobasal length (Cb.L), braincase width (Bc.W), muzzle width (Mu.W), zygomatic width (Zg.W), orbital diameter (Or.D), maxillary tooth‐row length (Mt.L), ramus mandibular height (Rm.H), mandible length (Md.L), mandibular tooth‐row length (Mdt.L) and coronoid height (Co.H) factors of Saanen goat with other goat breeds.

According to Tables 3 and 4, the mean ± standard deviation of Rm.H/Md.L, Mdt.L/Md.L and Or.D/Cb.L ratios in Saanen goats is smaller than the average of other breeds, but this decrease is not significant (*p *> 0.05). The mean ± standard deviation of Mt.L/Cb.L, Bc.W/Cb.L, Mu.W/Cb.L and Zg.W/Cb.L ratios in Saanen goats is greater than the average of other breeds, but this increase is not significant (*p* > 0.05) (Box plots 2 and 3).

**TABLE 3:** Ratio of ramus mandibular height (Rm.H)/mandible length (Md.L), mandibular tooth‐row length (Mdt.L)/Md.L, orbital diameter (Or.D)/condylobasal length (Cb.L), Mt.L/Cb.L, braincase width (Bc.W)/Cb.L, muzzle width (Mu.W)/Cb.L and zygomatic width (Zg.W)/Cb.L in some goat breeds.

Group	Rm.H/Md.L	Mdt.L/MdL	Or.D/Cb.L	Mt.L/Cb.L	Bc.W/Cb.L	Mu.W/Cb.L	Zg.W/Cb.L
Korean goat	38.79	43.34	57.76	61.86	73.50	8.04	60.08
Nigerian native goats	34.11	51.76	25.32	49.12	46.42	9.50	54.86
West Africa dwarf goat	44.64	60.11	18.00	30.94	32.70	5.43	45.76
Anglo Nubian goat	48.43	61.63	20.19	26.55	30.46	4.57	28.27
Red sokoto (Maradi) goats	51.59	64.27	21.23	27.47	32.57	4.71	30.64
Native goats in the Indian state of Mizoram	35.26	49.54	23.80	46.97	44.91	8.53	53.15
Black Bengal goats	35.29	50.09	24.56	47.54	43.42	8.66	53.78
Kagani goats	19.35	53.22	35.94	55.04	57.54	9.95	69.19
Abaza goats	38.67	55.41	30.26	54.16	51.86	10.01	60.52
European Saanen goat	36.31	51.30	25.75	48.38	47.58	10.05	55.18

**TABLE 4:** Mean ± standard deviation of ramus mandibular height (Rm.H)/mandible length (Md.L), mandibular tooth‐row length (Mdt.L)/Md.L, orbital diameter (Or.D)/condylobasal length (Cb.L), Mt.L/Cb.L, braincase width (Bc.W)/Cb.L, muzzle width (Mu.W)/Cb.L and zygomatic width (Zg.W)/Cb.L ratios in Sanen goats and other goat breeds.

Group	Rm.H/Md.L	Mdt.L/MdL	Or.D/Cb.L	Mt.L/Cb.L	Bc.W/Cb.L	Mu.W/Cb.L	Zg.W/Cb.L
Saanen	35.01 ± 2.75	51.30 ± 3.82	24.59 ± 2.29	49.43 ± 2.17	47.69 ± 3.59	9.82 ± 1.34	55.19 ± 2.01
The other goat breeds	38.27 ± 8.99	54.06 ± 6.36	28.38 ± 11.63	44.80 ± 12.25	46.09 ± 13.05	7.94 ± 2.21	51.14 ± 12.94

**Box plot 2:**
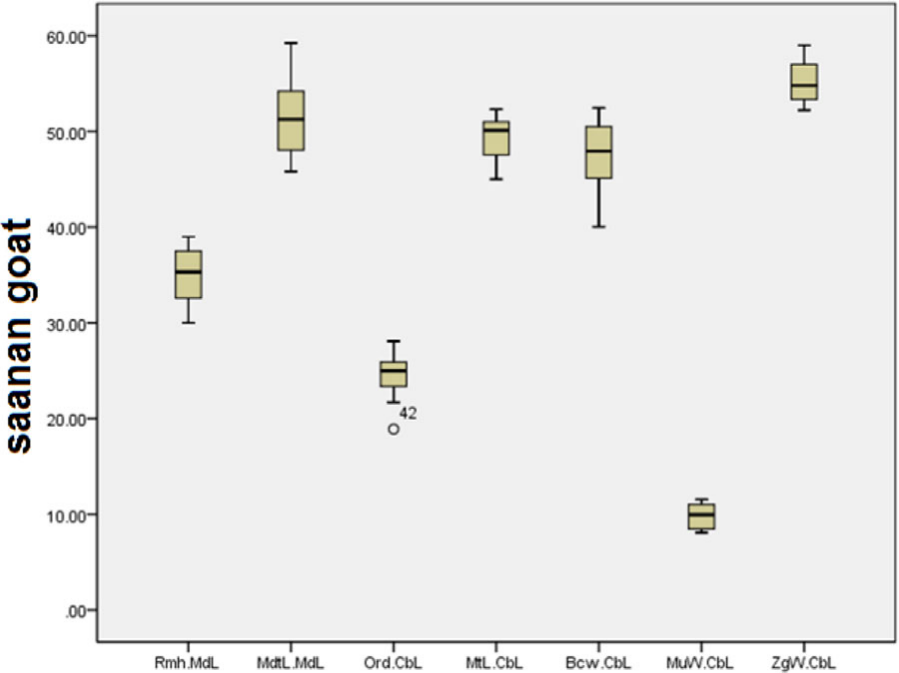
Mean ± standard deviation of ramus mandibular height (Rm.H)/mandible length (Md.L), mandibular tooth‐row length (Mdt.L)/Md.L, orbital diameter (Or.D)/condylobasal length (Cb.L), Mt.L/Cb.L, braincase width (Bc.W)/Cb.L, muzzle width (Mu.W)/Cb.L and zygomatic width (Zg.W)/Cb.L ratios in Saanen goats.

**Box plot 3:**
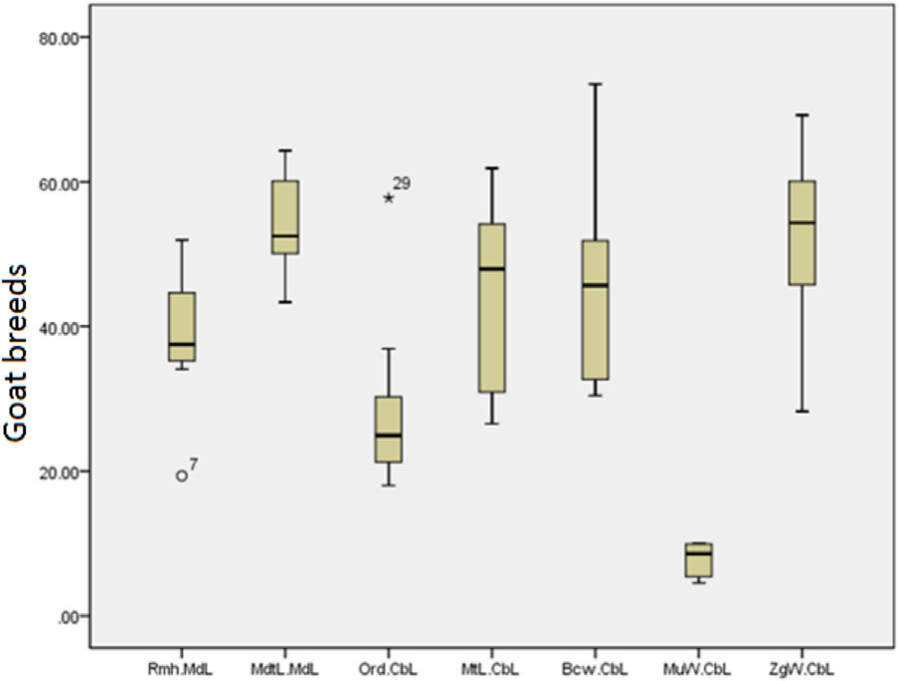
Mean ± standard deviation of ramus mandibular height (Rm.H)/mandible length (Md.L), mandibular tooth‐row length (Mdt.L)/Md.L, orbital diameter (Or.D)/condylobasal length (Cb.L), Mt.L/Cb.L, braincase width (Bc.W)/Cb.L, muzzle width (Mu.W)/Cb.L and zygomatic width (Zg.W)/Cb.L ratios in other goat breeds.

## 4 DISCUSSION

Several studies investigate the skeletal structure of the skull in native goats (Awaad et al., 2019; Goodarzi, 2014; Hykaj et al., 2012; Monfared & Sheibani, 2013). The size of various indexes was different in the current study compared with previous studies (Liu et al., 2018; Rupp et al., 2021). Compared to other animals, ruminants have wider muzzles. In one study, Korean native goats had an average Mu.W of 172.5 mm, whereas, in another study, Nigerian native goats had a mean Mu.W of 81 mm (Olopade & Onwuka, 2009; Yi et al., 1998). The mean of Mu.W in the Saanen goat was measured at 85.2 mm, making it smaller than native goats from Korea and larger than those from Nigeria. The coronoid process of the mandible is shorter and slightly curved in non‐ruminants while having a longer and stronger caudal curvature in ruminants (Subbaramaiah & Jagannatha, 2018). In this study, the mean Co.H of the Saanen goat was 27.8 mm, and its structure was similar to that of other ruminants. According to Olopade and Onwuka (2005), the Cb.L of Anglo‐Nubian breed goats was 215 mm and that of West African dwarf goats was 170 mm. In another similar study, Olopade (2018) reported that the Cb.L in red Sokoto (Maradi) goats was 202 mm, whereas the Cb.L in the present study was 86.6 mm, indicating that the Cb.L in Saanen goats was shorter than that of these breeds. In a study, Choudhary et al. (2020) observed that the Bc.W of native goats in the Indian state of Mizoram was 34.9 mm, and Uddin et al. (2009) found that the Bc.W of Mizoram was 32.7 mm. In the present study, Bc.W in Saanen goats was measured at 41.3 mm, which is higher than the figure reported for local goats in the Indian state of Mizoram and lower than that of Black Bengal goats. According to previous studies, Zg.W in West African dwarf goats, Kagani goats and Abaza goats has all been reported to be 77.8, 66.5 and 55.2 mm, respectively (Din et al., 2020; Dalga, 2020; Ngoran et al., 2019). However, in the current study, Zg.W in Saanen goats was 47.8 mm. As a result, Saanen goat appears to have the smallest Zg.W among these breeds. The Or.D in West Africa Dwarf, Anglo‐Nubian and red Sokoto goats has been reported 30.6, 43.3 and 24.6 mm, respectively (Olopade & Onwuka, 2005). We found that Or.D in Saanen goat was 21.3 mm, which was smaller than the Or.D of the mentioned breed. The results of Wang et al.’s (2021) tests on the skull of the European Saanen goat are compatible with our findings. They found that the Cb.L, Md.L and Mdt.L were 88.4, 63.7 and 1.32 mm, respectively. The skull was extended, and the facial region was relatively long. The palate was relatively wider, and the skull was also wide and short. The skull was extended and relatively long. Moreover, the extreme caudal part of the zygomatic arch was in an upper position.

But in the current study, the nasal bone was narrow and long. The premaxilla and incisive bones were closed to the cranial process. The lacrimal bone works as an independent bone in Saanen goats. The results disagree with previous studies that showed lacrimal bone does not work as an independent bone (Vaccari et al., 2009). The premaxilla bone and incisive teeth formed a small part of the palate. In all goats, the transverse crest was positioned at the extreme of the hard palate, and the crest is direct. Other studies have reported a curved crest in Saanen goats (Palombo & Villa, 2003). One of the prominent points in the ventral view of the Saanen goat skull was the presence of foramina in the hard palate. It was reported that cranial foramens are carriers for palate nerves (Ashdown & Done, 2001). The orbit region of temporal bones was described by previous studies and reported frontal section as an independent region in European young goats (Ortego et al., 2011). The zygomatic arch was completely distinct. Seemingly, the former section of the arch is distinguishable from the maxilla, and it was also seen in the current study. The jugular bone was limited to the medium region of the arch. The tympanic part of the ventral surface was grown in the goats, and the tympanic bulla was distinguishable which is in agreement with the current study (Nemeth et al., 2017). The most important properties of the ventral position of the skull were the processes. It means that typical and external processes are found in all goats. The external process is formed from the caudal extreme of the palate bone and sphenoid bone (Wilson, 2020). The processes were also found in the current study. Haykaj et al. (2012) investigated European goats and reported the changes in the skull and nasal bone indexes which confirm their applied importance. They also reported process parietalis ossis frontalis as an applied part. Significant differences between goats in altitudes and those reared in plane regions are reported (Ayele et al., 2016).

The results of Cb.L, braincase breadth, rostrum breadth, zygomatic breadth, Or.D, the height of ramus mandibulae, length of mandible, Mdt.L and Co.H are in agreement with previous studies (Erdal et al., 2019). The results for the radiographic section are also parallel with other data for radiological section (Lampignano & Kendrick, 2021; Silverman & Tell, 2021). In the radiographic section, the zygomatic arch was seen which is formed from zygomatic and temporal bone which is similar to other ruminants (Wang et al., 2021). It is formed from maxilla, jugular bone and temporal bones which are seen in dorsoventral and caudoventral positions. It was not seen distances among incisors, canines and molar teeth.

## 5 CONCLUSIONS

In conclusion, this study was conducted to investigate radiological and anatomical features of the skull bone in adult Saanen goats and showed similarities between Saanen goats and other goats. The size of various indices in the present study was different compared to previous studies, so that the average Mu.W, Cb.L and Zg.W of Saanen goats were smaller than other goat breeds. The lacrimal bone was an independent bone, and one of the prominent points in the ventral view of the Saanen goat skull was the presence of foramina in the hard palate. The precise results acquired in this study can be utilized to interpret the findings and make clinical decisions about the normal and abnormal size of the bones that make up the skulls of the Saanen goats.

## AUTHOR CONTRIBUTIONS

*Conceptualization; data curation; formal analysis; funding acquisition; investigation; methodology; project administration; resources; software; supervision; validation; writing – original draft; writing – review and editing*: Siamak Alizadeh. *Investigation; resources; writing – original draft*: Pourya Kamfar. *Formal analysis; investigation*: Mohammadreza Hosseinchi.

## CONFLICT OF INTEREST STATEMENT

All authors declare no conflicts of interests.

## FUNDING INFORMATION

There are no funders to report for this submission.

### ETHICS STATEMENT

This study does not present any ethical concerns.

### PEER REVIEW

The peer review history for this article is available at https://publons.com/publon/10.1002/vms3.1435.

## Data Availability

The data that support the findings of this study are available from the corresponding author upon reasonable request.
